# With a Little Help from My Cell Wall: Structural Modifications in Pectin May Play a Role to Overcome Both Dehydration Stress and Fungal Pathogens

**DOI:** 10.3390/plants11030385

**Published:** 2022-01-30

**Authors:** Ariana D. Forand, Y. Zou Finfrock, Miranda Lavier, Jarvis Stobbs, Li Qin, Sheng Wang, Chithra Karunakaran, Yangdou Wei, Supratim Ghosh, Karen K. Tanino

**Affiliations:** 1 Department of Plant Sciences, University of Saskatchewan, Saskatoon, SK S7N 5A8, Canada; adf472@usask.ca (A.D.F.); sheng.wang@usask.ca (S.W.); 2 Advanced Photo Source, Lemont, IL 60439, USA; finfrock@anl.gov; 3 Canadian Light Source, Saskatoon, SK S7N 2V3, Canada; Miranda.Lavier@lightsource.ca (M.L.); jarvis.stobbs@lightsource.ca (J.S.); chithra.karunakaran@lightsource.ca (C.K.); 4 Department of Biology, University of Saskatchewan, Saskatoon, SK S7N 5E2, Canada; li.qin@usask.ca (L.Q.); yangdou.wei@usask.ca (Y.W.); 5 Department of Biochemistry, Microbiology and Immunology, University of Saskatchewan, Saskatoon, SK S7N 5E5, Canada; 6 Department of Food and Bioproduct Sciences, University of Saskatchewan, Saskatoon, SK S7N 5A8, Canada; supratim.ghosh@usask.ca

**Keywords:** cell wall, pectin, calcium, boron, homogalacturonan, rhamnogalacturonan II, *Botrytis cinerea*, *Colletotrichum higginsianum*, dehydration, *Allium* and X-ray microscopy

## Abstract

Cell wall structural modifications through pectin cross-linkages between calcium ions and/or boric acid may be key to mitigating dehydration stress and fungal pathogens. Water loss was profiled in a pure pectin system and in vivo. While calcium and boron reduced water loss in pure pectin standards, the impact on *Allium* species was insignificant (*p* > 0.05). Nevertheless, synchrotron X-ray microscopy showed the localization of exogenously applied calcium to the apoplast in the epidermal cells of *Allium fistulosum*. Exogenous calcium application increased viscosity and resistance to shear force in *Allium fistulosum*, suggesting the formation of calcium cross-linkages (“egg-box” structures). Moreover, *Allium fistulosum* (freezing tolerant) was also more tolerant to dehydration stress compared to *Allium cepa* (freezing sensitive). Furthermore, the addition of boric acid (H_3_BO_3_) to pure pectin reduced water loss and increased viscosity, which indicates the formation of RG-II dimers. The *Arabidopsis* boron transport mutant, *bor1*, expressed greater water loss and, based on the lesion area of leaf tissue, a greater susceptibility to *Colletotrichum higginsianum* and *Botrytis cinerea*. While pectin modifications in the cell wall are likely not the sole solution to dehydration and biotic stress resistance, they appear to play an important role against multiple stresses.

## 1. Introduction

As the threat of climate change rages on, the frequency of dehydration stress and the risk posed by fungal pathogens is on the rise. These challenges are further complicated by the sessile nature of plants, which must survive a range of a combination of abiotic and biotic stresses. The cell wall serves as a barrier against stress, in addition to providing critical mechanical and structural support to the plant [1,2]. The ability of the cell wall to function in a range of mechanisms is dependent on the various components of the primary cell wall, including cellulose, hemicellulose, proteins and pectin [3]. Except for grasses, pectin is one of the most predominant components, comprising 30 to 50% of the cell wall dry matter [3]. Therefore, the composition of pectin is integral to the growth, morphology and development of the cell well, as well as in its ability to defend the plant against stress [4].

Pectin, the most structurally complex polysaccharide found in nature, is a five-member family currently considered to include: (1) Homogalacturonan (HG); (2) Rhamnogalacturonan I (RG-I); (3) Rhamnogalacturonan II (RG-II); (4) Xylogalacturonan (XGA); and (5) Apiogalacturonan (AP) [4]. HG and RG-II account for approximately 65% and 10% of all pectin, respectively, and have the unique ability to form more complex structures through cross-linkages to other elements [4,5,6].

Under the prevailing view, calcium ions form cross-linkages to carboxylate ions in demethylesterified galacturonic acid residues of HG [5,7]. The demethylesterifcation of HG occurs by way of pectin methylesterases (PMEs) [7] and pectin methylesterase inhibitors (PMEIs) provide negative feedback to PMEs [7]. In comparison, RG-II forms cross-linkages with boric acid, creating RG-II dimers [8]. These borate–diol ester cross link a boron atom and the apiosyl residue of side chain A in RG-II [8]. The formation of these cross-linkages maintains structural stability by reducing porosity while enhancing tensile strength and bound water in the cell wall fraction [9,10,11,12,13,14,15,16]. Increasing the quantity of bound water within the cell wall maintains tissue hydration and turgor pressure in addition to increasing cell wall rigidity [14]. Debra Mohnen’s group recently proposed a new model for pectin structure consisting of a family of glycans (homoglycans, heteroglycans and proteoglycans) [17] and therefore, aspects of the current cell wall models may need to be reconsidered as more information emerges.

HG and RG-II have also been implicated as key forms of pectin in mitigating the resistance to desiccation and drought stress in several species, such as green algae and wheat cultivars [18,19,20,21,22,23]. This is likely the result of pectin forming hydrated gels, which in turn may limit damage during dehydration stress [19]. The degree of pectin methylation has also been linked to their ability to hold water, with reduced methylation increasing the water holding capacity of pectin [12]. Willats et al. (2001) also found the addition of calcium to pectin gels influences the water holding capacity, presumably as a result of “egg-box” structures [12].

The analysis of the *MUR1* gene has further implicated RG-II dimers as an important aspect of the tolerance to freezing stress, tied to dehydration stress resistance [24]. Despite these findings, there is a need for greater research regarding the role calcium and boron on the structure–function of the cell wall in relation to dehydration stress resistance.

Previous research has additionally highlighted the important role that pectin plays in allowing the cell wall to function as a barrier to *Botrytis cinerea*, a necrotrophic pathogen, and *Colletotrichum higginsianum*, a hemibiotrophic pathogen. Lionetti et al. (2007) observed increased resistance to *B. cinerea* in *Arabidopsis* as a result of the over-expression of PMEI1 and PMEI2 [25]. Other PMEIs have also been reported to increase plant resistance to *B. cinerea* [26]. Interestingly, PMEs play a beneficial role in the immunity against *B. cinerea*, despite the opposing nature of PMEIs and PME [27]. More generally, pectin has been identified as a main target during a *B. cinerea* infection [28]. Petrasch et al. (2019) reported that, during the process of a *B. cinerea* infection, the pathogen heavily degrades the cell wall and, in particular, pectin [28].

PMEIs also play a role in the immune response during a *C. higginsianum* infection [29]; however, research within this area is limited. A study conducted by Engelsdorf et al. (2017) found *Arabidopsis pmei6-2* mutants had reduced susceptibility to *C. higginsianum*. Reduced susceptibility may be indicative of a connection between the establishment of a *C. higginsianum* infection and pectin content [29]. Nonetheless, further exploration into the role of PMEIs and *C. higginsianum* is required.

Boron application has also been shown to play a positive role in *B. cinerea* infections in a variety of species [30,31]. Qin et al. (2010) found boron application reduced the germination of *B. cinerea* spores, reduced germ tube elongation and mycelial spread in table grapes [30]. When paired with boron, calcium increased resistance to *B. cinerea* in strawberry plants [31]. In addition, while there is no apparent research on the impact of boron on *C. higginsianum*, borate was found to inhibit the germination of *Colletotrichum gleosporioides* spores in mangos [32,33].

*Allium fistulosum* and *Allium cepa* were selected based on their contrasting freezing-induced dehydration stress tolerance. *A. fistulosum* is extremely tolerant to freezing stress, withstanding temperatures as low as −40 °C, while *A. cepa* lacks this ability [34,35]. Both species have easy to peel epidermal cell layers with a large cell size, making them an ideal species for this investigation. *Arabidopsis* was selected based on the wide range of genotypic mutant lines available and its close genetic relationship to *Brassica rapa*.

Since plants are often exposed to multiple stresses in the field, this study aims to gain a further understanding into the influence of calcium, boron and PMEI on pectin cross-linkages, and in turn, on the ability for the cell wall to act as a barrier against both water loss and fungal pathogens. We hypothesized the application of calcium and/or boron results in cell wall structural changes and increased resistance to both abiotic and biotic stress in *Allium* species and *Arabidopsis thaliana.*

## 2. Method and Materials

### 2.1. Plant Material and Growth Conditions

*A. fistulosum* seeds and Yellow Sweet Spanish *A. cepa* seeds (Early’s Farm & Garden Center, Saskatoon, SK, Canada) were grown in the Agriculture Greenhouses, University of Saskatchewan (Saskatoon, SK, Canada) in 6″ pots containing Sunshine Mix No. 4 (Sungro Horticulture Canada Ltd. Seba Beach, AB, Canada) at approximately 25/22 °C (day/night) under natural light supplemented with high pressure sodium lights (17 h photoperiod, average 600 μmol m^−2^s^−1^). Watering (City of Saskatoon, SK, Canada, water) was applied every second day during the spring/summer months and every third day during the fall and winter months with 200 mL of 20–20–20 (NPK) fertilizer (150 g L^−1^) (Plant Prod, Brampton, ON, Canada) weekly during the spring/summer months and twice weekly during the fall/winter months. The experiment was arranged in a randomized complete block design across the bench.

In addition, five *A. thaliana* genotypes were analyzed: three boron transporter mutants (*nip5;1-1* (SALK_122287C), *nip6;1-2* (SALK_046323C) and *bor1-3* (SALK_037312)), a pectin methylesterase inhibitor over-expression mutant (*p35S::PMEI5*) and a wild-type (Col-0) line, respectively [36,37,38,39]. The three boron mutants were obtained from the *Arabidopsis* Biological Resource Centre (ABRC) (Columbus, OH, USA). The transgenic line overexpressing PMEI5 driven by the Cauliflower mosaic virus (CaMV) 35S promoter was provided courtesy of Kerstin Müller [39]. Lines were genotyped according to Edwards et al. (1991), with some modifications [40,41]. Gene specific primers were ordered from Integrated DNA Technologies (Coralville, IA, USA) (Table S1).

*Arabidopsis* plants were grown in the Agriculture phytotron (Conviron, Winnipeg, MB, Canada) under 20 °C constant temperature, 50% RH, 16 h photoperiod, with an irradiance of 150 ± 10 m^−2^s^−1^, and watered every second day (City of Saskatoon, SK, Canada). Two g/L fertilizer (20–20–20 NPK) was applied weekly.

### 2.2. Forms of Calcium and Boron Application

Calcium treatments of 100 mL of a 0.05 M CaCl_2_ (Fisher Scientific, Watham, MA, USA) were applied every second day for four weeks as a soil drench. For pectin solutions prepared with calcium or boron, 0.05 M of CaCl_2_ or H_3_BO_3_ were added directly during the preparation of the pectin solution to ensure homogeneity.

### 2.3. Rheology

Two types of pectin were examined, TIC Pretested Pectin HM (69–75% methylation) [42] slow set (standardized with dextrose) (TIC Gums, White Marsh, MA, USA) and GENU BETA pectin (55% methylation) [43] (standardized with EU non-GM beet sucrose) (CP Kelco Atlanta, GA, USA). The HM pectin is derived from citrus pectin and as a result has a high HG content and a lower RG content, making it similar to the pectin found within the cell wall of *Allium* spp. [44,45]. GENU BETA pectin is produced from sugar beets, and as such it has a higher RG-II content (~5% compared to 0.6%), making it more like the pectin found within *Arabidopsis*. Therefore, HG pectin was utilized as a proxy for *Allium*, while GENU BETA pectin was selected as a proxy for *Arabidopsis*.

Pectin viscosity was measured using the AR G2 rheometer (TA Instruments, New Castle, DE, USA) with the 40 mm smooth acrylic geometry. Measurements were taken at 20 °C, 12 °C and 4 °C. Viscosity was also measured throughout the temperature ramp. Prior to the first set of measurements at 20 °C, the sample was conditioned to 20 °C with a soak time of 120.0 s^−1^ and a duration of 60.0 s^−1^. Viscosity measurements were then taken for 300.0 s^−1^ with a shear rate of 0.11 s^−1^. The sampling rate for the measurements taken was 10 s^−1^/pt. The temperature was then decreased to 12 °C at a rate of 5.0 °C/min, with the shear rate remaining constant at 0.11 s^−1^. The sample was then conditioned with the same soak time and duration as stated above. Viscosity measurements were also conducted under the same conditions previously outlined. The temperature was decreased to 4 °C and measurements of viscosity were performed as previously described above. The experiment was repeated three independent times with four replicates of each treatment group per trial (*n* = 12).

### 2.4. Texture Analysis

The force required to shear *A. fistulosum* sheaths was evaluated using the 10-blade Allo–Kramer shearing compression cell, attached to the TMS-Pro Texture Press (Food Technology Corp., Sterling, VA, USA) in the Shand lab at the University of Saskatchewan. Calcium-treated plants received 100 mL soil drench of a 0.05 M CaCl_2_ solution every second day for four weeks. Each measurement was performed using three 4 cm sections of *A. fistulosum* sheaths, from the youngest leaf with the most developed sheath. The ligule and the epidermal cell layer were left intact. The full-scale load was 1000 N, the crosshead speed was 500 mm min^−1^. The shear force (in Newtons) required to shear 1 g of fresh sample was calculated using Equation (1) [46]. The shear force was measured in N g^−1^ [46]. The experiment was repeated three independent times, with four replicates per trial (*n* = 12).

(1) $$Force\;Required\;to\;Shear\;Allium\;Sheaths=\frac{peak\;shear\;force\;\left(N\right)}{weight\;of\;sheaths\;\left(g\right)}\;$$

Equation (1). Force required to shear *Allium fistulosum* sheaths in N g^−1^.

### 2.5. Water Loss in Pure Pectin Standards

TIC Pretested Pectin HM (69–75% methylation) slow set (standardized with dextrose) was used as a model for pectin within *Allium* and GENU BETA pectin (55% methylation) (standardized with EU non-GM beet sucrose) was a model for *Arabidopsis* pectin. Treatments consisted of pectin solutions at concentrations of 4% and 8%, with and without 0.05 M of CaCl_2_ or 0.05 M of H_3_BO_3_. Overall, 12 different pectin solutions were examined with 4 replications per pectin solution treatment, and the experiment was repeated 3 times.

Gravimetric water loss on an analytical balance was recorded at hourly intervals over 0–6 h in the various pectin solutions. This timeline was selected following preliminary experiments. Approximately 1 g of each of the pectin solutions was evenly pipetted into Petri dishes from the center point. Throughout the course of the dehydration experiment, the lids were kept off the Petri dishes to allow for evaporation. The temperature remained constant at 23 °C, with a relative humidity of ~22%. The percentage water loss was measured as per Equation (2).

(2) $$Percentage\;Water\;Loss=\left(1-\left(\;\frac{T0\;weight}{T\left(x\right)weight}\right)\times 100\right)$$

Equation (2). Percentage water loss in pure pectin standards. Variable “*T*” is equal to the mass of the plant tissue at a specified time point.

### 2.6. Allium Dehydration Stress Tolerance

Dehydration tolerance was measured by first analyzing the percentage water loss over 16–18 h using 4 cm sections of *A. fistulosum* and *A. cepa* sheaths obtained from the youngest leaf with the most developed sheath. Both ends of the sheath were sealed with Vaseline^®^ and weighed to obtain a T0 weight. The sheaths were then dehydrated under dark conditions at 23 °C and ~33% RH. Weights were recorded at 16 h and 18 h. Following dehydration, the sheaths were wrapped in moist Kim Wipes and placed in 50 mL Falcon tubes with the lid for 24 h. The percentage water loss was measured and calculated (Equation (2)).

Following dehydration, viability was assessed based on the presence/absence of protoplasmic streaming and staining using fluorescein diacetate (FDA). Epidermal cell layers were peeled from the sheath prior to viability analysis. Protoplasmic streaming was observed using a digital LEICA DM4 B microscope (Wetzlar, Germany) at 40× with a LEICA DFC7000 T camera (Wetzlar, Germany). Protoplasmic streaming was quantified as a percentage of viability across the epidermal cell layer (Equation (3)). The same microscope was used to visualize epidermal cell layers at 40× following FDA staining.

(3) $$Percent\;Cell\;Viability=\frac{number\;of\;cells\;with\;protoplasmic\;streaming\;}{total\;number\;of\;cells\;}$$

Equation (3). Percent cell viability based on protoplasmic streaming. Total cell count was measured by counting the number of cells within the frame at the 40× objective.

### 2.7. Arabidopsis Dehydration Stress Tolerance

Tolerance to dehydration stress was assessed by measuring percentage water loss every hour over 10 h in the total above ground biomass of the various two-week-old genotypes. An initial weight was recorded at 0 h and then every 2 h until 10 h. The percentage water loss was measured and calculated (Equation (2)). The samples were kept under dark conditions at 23 °C and ~33% RH. Prior to dehydration, the severed end of the shoot was sealed using Vaseline^®^. Following dehydration, plants were rehydrated in 2 mL sepia toned bottles with 100 µL of dH_2_O.

Viability following dehydration was assessed using electrical conductivity (Twin Compact Meter, Horiba, Japan) by first adding 1000 µL of dH_2_O to the bottles ~24 h following rehydration and then placing the bottles on the shaker for ~19 h at 23 °C. A second total electrical conductivity measurement was then taken after placement in a 100 °C dry bath for 10 min and then vortexed. Percentage electrolyte leakage (µS/cm) was measured and calculated (Equation (4)) [47].

(4) $$Perecent\;Electrolyte\;Leakage=\frac{\left(\left(\frac{Pre-Boil\;Conductivity\;Value}{Post-Boil\;Conductivity\;Value\;}\right)\times 100\right)}{T0\;weight}$$

Equation (4). Percent electrolyte leakage in *Arabidopsis thaliana* above ground biomass following dehydration, where *T*0 was the time at 0 h.

The experiment was conducted under dark conditions to minimize the amount of water lost through open stomata. Stomatal closure was confirmed using the Suzuki Universal Micro-Printing (SUMP) method [48]. SUMP discs and SUMP liquid were used to take imprints of stomata, while a digital LEICA DM4 B microscope (Wetzlar, Germany) at 40× with a LEICA DFC7000 T camera (Wetzlar, Germany) attached assessed the imprints.

### 2.8. Calcium Localization

Calcium was spatially localized within single epidermal cell layers obtained from *A. fistulosum* using X-ray microscopy. Plants were grown and treated with calcium chloride as outlined above. The peeled epidermal layers were laid flat on Kapton tape and were used for data collection at the Advanced Photon Source (APS) beamline (20-ID: Sector 20—Insertion Device Beamline) (Lemont, IL, USA). Characteristics of the X-ray beam: incident energy—10 keV; sample scanning area—160 µm × 160 µm; scanning step size—1 µm; and dwell time—10 milliseconds per pixel. A total of 10 vertical scan maps were collected at a depth of every 2 µm into the sample [49].

Two-dimensional images were processed using PyMCA to fit the normalized average spectra of all points on the map and to generate the calcium distribution map (Version 5.3.1) [50]. OriginPro (Northhampton, MA, USA) was then used to plot the calcium map and to increase image quality by eliminating the saturated pixels. False color maps were produced using rainbow color scheme in which the blue-to-red color gradient shows low-to-high relative calcium concentrations.

The completed images were then analyzed using the “histogram” feature on ImageJ (Version 1.53a) to gain a greater understanding into differences in RGB pixels between control and calcium-treated epidermal cell layers.

### 2.9. Colletotrichum higginsianum Infection

*C. higginsianum* Sacc culture, isolate IMI349061, originating from *Brassica rapa* (CABI Bioscience), was prepared as outlined in Liu et al. (2010) and Liu et al. (2007) [51,52]. Spores were suspended in ddH_2_O, with a final concentration of 10^6^ spores/mL. Plants were inoculated by applying multiple single 1 µL droplets of inoculant to the oldest leaf, avoiding the mid-vein.

Throughout the course of infection, leaves were maintained on moist filter paper in Petri dishes kept under a 16–8 h/light–dark period (150 ± 10 µmol m^−2^s^−1^) during the first 2 and last 2 days of the period of infection, at the third day the plates were transferred to complete darkness. Complete darkness was used to promote germination of the appressorium, while the leaves were transferred back to a day/night light cycle on days 4–5 to prevent chlorosis at approximately 20 °C throughout.

Infection was then evaluated every 24 h based on the percent total leaf area infected (Equation (5)). The lesion area was measured using ImageJ (Version 1.53a).

(5) $$Percent\;leaf\;area\;infected=\left(\frac{Lesion\;area\;\left(cm^{2}\right)\;at\;T\left(x\right)}{Total\;leaf\;area\;\left(cm^{2}\right)\;at\;T\left(x\right)}\right)\times 100$$

Equation (5). Percent leaf area infected by *Botrytis cinerea* and *Colletotrichum higginsianum*, where *T*(*x*) was the time point of interest.

### 2.10. Botrytis cinerea Infection

*B. cinerea* grown on potato dextrose agar (PDA) for 7 days at 22 °C was used to inoculate four-week-old *A. thaliana* leaves [53]. The leaves were inoculated using a solution containing *B. cinerea* mycelium suspended in ddH_2_O, with a final concentration of 10^6^ spores/mL. Three 1 µL droplets of the mycelium containing solution were applied to one half of the leaf, while three 1 µL droplets of water were applied to the other half. Caution was taken to avoid the leaf mid-vein. Throughout the course of infection (120 h), the leaves were maintained on moist filter paper within sealed Petri dishes and kept the dark at 23 °C throughout the course of infection.

The infection was evaluated based on the percent total leaf area infected (Equation (5)).

### 2.11. Statistical Analysis

Analyses related to shear force, water loss, protoplasmic streaming, electrolyte leakage, and fungal pathogens were performed using ANOVA tests, with Tukey’s tests as a method of post hoc analysis (*p* < 0.05). Two-tailed two-sample *t*-tests (*p* < 0.05) analyzed calcium localization according to differences in RGB pigmentation and differences in the intensity of green pixels following FDA staining.

Rheological results were analyzed using generalized additive models (GAMs). Models were initially fit based on each fixed explanatory variable separately, prior to fitting models at all fixed explanatory variables together. Data were log-transformed. GAMs were generated using the gam function from the “mgcv” package [54,55]. The Akaike information criterion (AIC) score was used when building models to select for the optimum model. Function gam.check was used for model checking. GAMs were analyzed using ANOVA, with F-tests. Figures of each GAM were individually created for each of the pectin solutions using function “plot.gam”, in addition to the packages “mgcViz” and “rgl” [56,57]. The package “devtools” was used to download the color palette, and “inauguration_2021” was used for coloration figures [58].

All statistical analyses were performed with the RStudio statistical software (Version 1.2.5033). In addition to the previous packages mentioned, “ggplot” and “ggplot2” were also used [58].

## 3. Results

**Figure 1:**
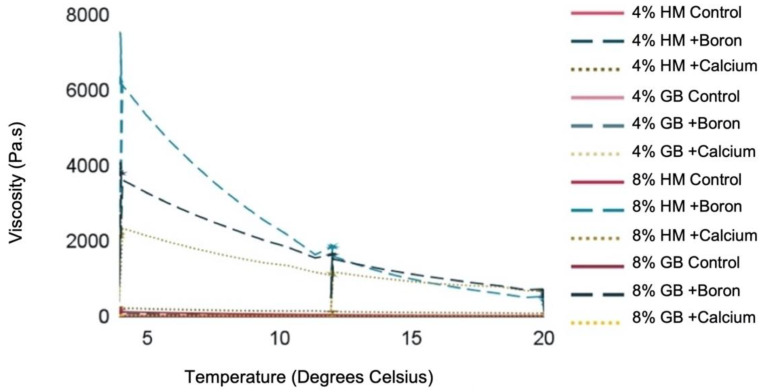
Change in viscosity (Pa.s) as temperature (°C) increased. Viscosity was analyzed across two concentrations of pectin (4% or 8%), two types of pectin (high methylated citrus pectin or GENU BETA sugar beet pectin) and with either calcium (0.05 M CaCl_2_), boron (0.05 M H_3_BO_3_) or no additional element.

Pectin viscosity significantly (*p* < 0.05) increased under the application of boron alone in both HM pectin and GENU BETA sugar beet (GB) pectin (Figure S1, Table S2). Boron had a more pronounced impact on 8% HM pectin viscosity at 5 °C, increasing the viscosity of HM pectin to just over 6000 Pa.s 5 °C compared to 8% GB pectin with boron, which had an average viscosity of less than 4000 Pa.s at 5 °C (Figure 1). Boron also had a more notable impact on HM pectin at 12 °C, with the trend only changing at 20 °C, when boron increased GB pectin viscosity to levels greater than HM pectin viscosity (Figure 1). Interestingly, calcium application alone did not have a significant effect on pectin viscosity (Table S2). As outlined in Section 2.2, calcium and boron were added directly to the pectin solution. However, calcium did have a significant effect on pectin viscosity when its influence was considered in combination with increasing pectin concentration and temperature (Table S2). The effect of boron on pectin viscosity remained significant when its effect was considered in combination with increasing pectin concentration and temperature (Table S2).

**Figure 2:**
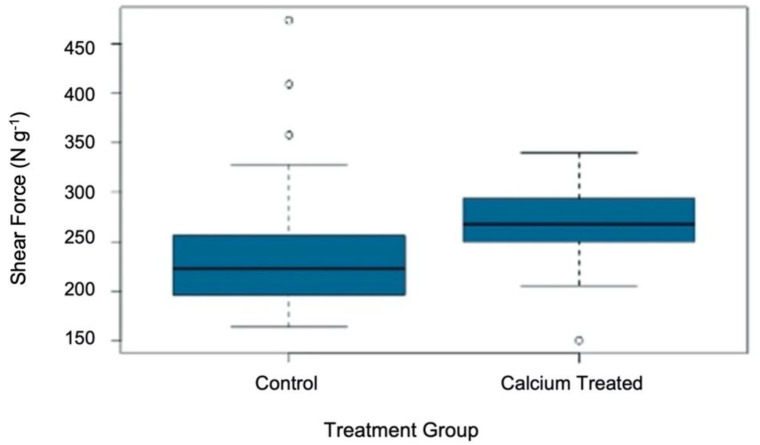
The effect of calcium application on the force (N g^−1^) required to shear through *Allium fistulosum* sheaths. Plants treated with calcium received 100 mL of a 0.05 M CaCl_2_ solution every second day. See Table S3 for statistical analysis.

The analysis of the force required to shear *A. fistulosum* sheaths revealed the application of calcium (applied as a soil drench) resulted in a significantly (*p* < 0.05) higher Allo–Kramer shear force (265.0 N g^−1^), compared to *A. fistulosum* plants that had not received calcium (210.6 N g^−1^) (Figure 2). This increase in shear force is representative of an increased toughness within the sheaths. Toughness is a mechanical property that describes the ability for a material to resist fracture [59,60]. The toughness of a material is influenced by both its strength and ductility [59,60].

**Figure 3:**
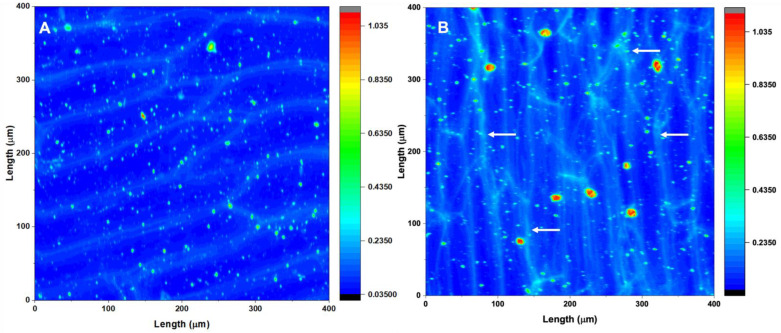
2D Calcium map obtained using the 20-ID beamline at the Advanced Photon Source, Lemont, IL, and the X-ray microscopy technique. Calcium concentration is represented by color changes within the map, with darker blue indicating a lower concentration of calcium to red indicating a higher concentration of calcium. White arrows point to sample areas where calcium appears to localize to the apoplast. (**A**) Single *Allium fistulosum* epidermal cell layer obtained from a control plant without calcium treatment. (**B**) Single *Allium fistulosum* epidermal cell layer obtained from a plant treated with a soil drench of 100 mL of a 0.05 M CaCl_2_ every second day for four weeks.

Calcium was spatially localized within *A. fistulosum* epidermal cell walls (Figure 3). X-ray microscopy imaging showed that the exogenous application of calcium to *A. fistulosum* increased the concentration of calcium within the cell layer, with the additional calcium primarily localizing to the apoplast (Figure 3). The spatial localization of calcium to the apoplast is evident in Figure 3B, where epidermal layer cell walls can be observed because of increased calcium concentrations in the apoplast (more intense lighter blue color). This calcium appears to be primarily distributed to the radial cell walls. These areas of interest are identified using white arrows. Small flecks of white, light blue and green are also visible across both Figure 3A,B. These, in addition to red flecks, which are more pronounced on Figure 3B, represent calcium contamination and are not related to structures within the cell layer.

**Figure 4:**
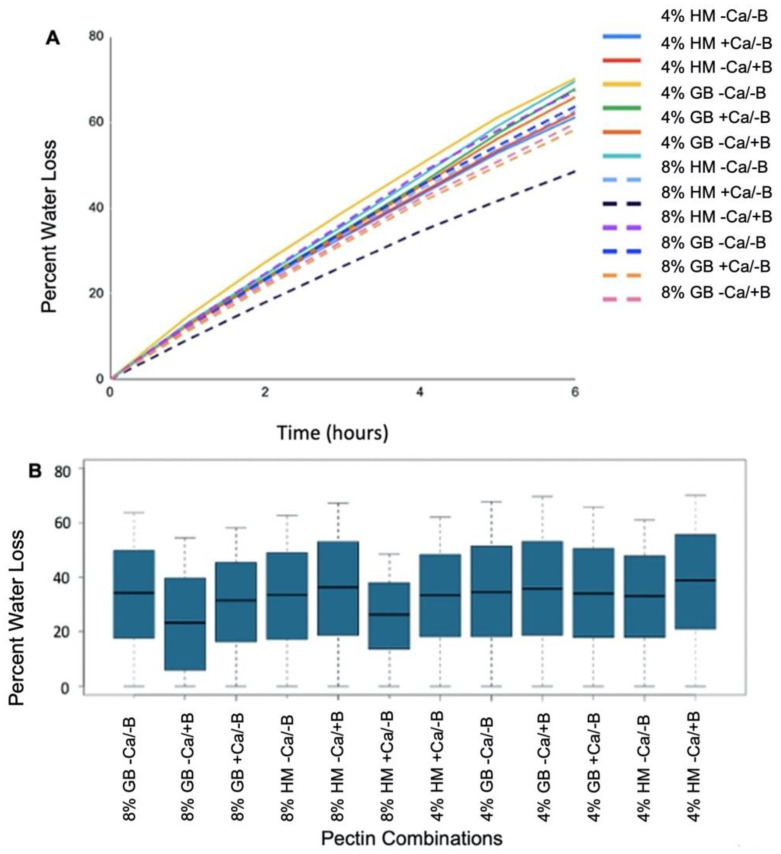
(**A**) Percent water loss over 6 h over a 6 h period for the 12 pectin solutions of interest. Amongst the 12 solutions, there are two concentrations (4% or 8%), two pectin types (high methylated (HM) citrus pectin or GENU BETA (GB) (sugar beet) pectin), and the addition of calcium (Ca) (0.05 M CaCl_2_), boron (B) (0.05 M H_3_BO_3_) or no additional element. (**B**) Boxplot of average percent water loss over a 6 h period for the 12 pectin solutions of interest. Amongst the 12 solutions, there are two concentrations (4% or 8%), two pectin types (high methylated (HM) citrus pectin or GENUA BETA (GB) (sugar beet) pectin), and the addition of calcium (Ca) (0.05 M CaCl_2_), boron (B) (0.05 M H_3_BO_3_) or no additional element. See Tables S5 and S6 for statistical analysis.

In addition to increasing the toughness of *A. fistulosum* sheaths, calcium application significantly (*p* < 0.05) reduced percentage water loss, as did increasing pectin concentrations (Figure 4B). An 8 percent high methylated citrus pectin with calcium (proxy for *Allium*) lost the least amount of water after 6 h (48.5%), while 8% GENU BETA pectin with calcium (proxy for *Arabidopsis*) had a water loss of 58.3% after 6 h (Figure 4A). However, the effect of calcium in mitigating dehydration stress in *Allium* species tissue was not significant (*p* > 0.05) (Table S4).

**Figure 5:**
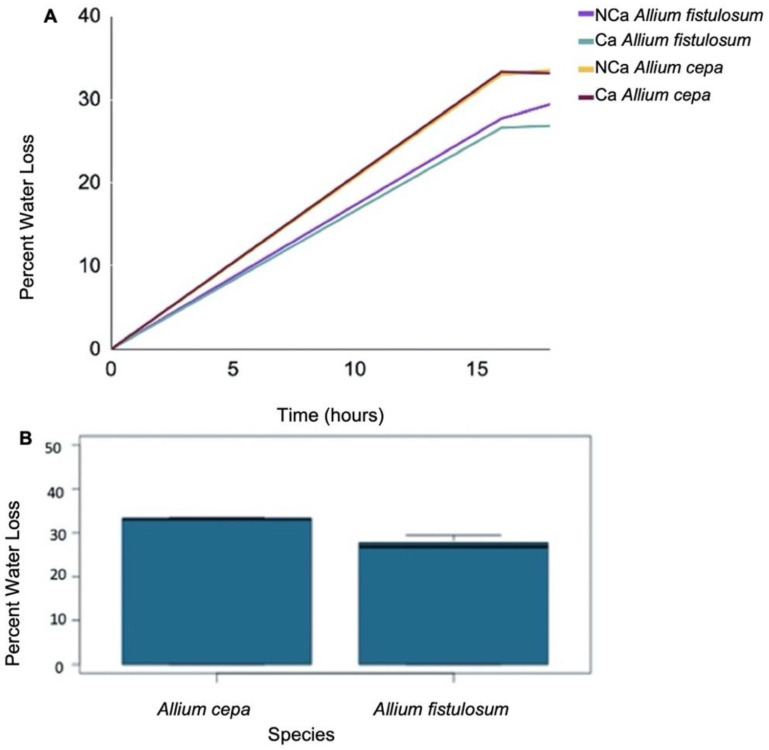
(**A**) Percent water loss over 16–18 h in *Allium fistulosum* and *Allium cepa*. Plants treated with calcium (CA) received 100 mL of a 0.05 M CaCl_2_ every second day for four weeks. Control plants (NCA) did not receive calcium. (**B**) Boxplot showing average percent water loss over 16–18 h in *Allium fistulosum* and *Allium cepa* sheaths. Each bar represents both control and calcium treated plants combined. See Table S4 for statistical analysis.

The freezing-tolerant *A. fistulosum* was also significantly (*p* < 0.05) more resistant to dehydration stress compared to the freezing-sensitive *A. cepa* (Figure 5B). After 17 h, *A. fistulosum* sheaths had a percentage water loss of 27.1%, while sheaths obtained from *A. cepa* lost 33.1% water (Figure 5A). *A. fistulosum* continued to lose less water as the period of dehydration was extended from 16 h to 18 h, retaining 5.2% more water compared to *A. cepa* (28.1% compared to 33.3%) (Figure 5A).

**Figure 6:**
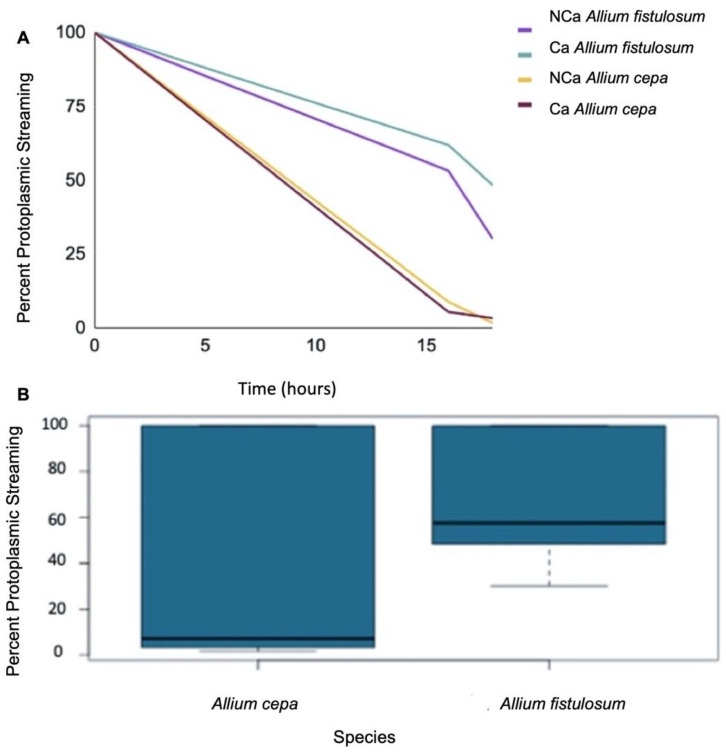
(**A**) Percent protoplasmic streaming in *Allium fistulosum* and *Allium cepa* epidermal cell layers following dehydration over 16–18 h. Plants treated with calcium (CA) received 100 mL of a 0.05 M CaCl_2_ every second day for four weeks. Control plants (NCA) did not receive calcium. (**B**) Boxplot showing percent protoplasmic streaming for *Allium fistulosum* and *Allium cepa*, following a 16–18 h dehydration period. Each bar represents both control and calcium treated plants combined. See Table S7 for statistical analysis.

In addition, epidermal cell layers from dehydrated and subsequently rehydrated *A. fistulosum* sheaths had a significantly (*p* < 0.05) higher percentage viability based on protoplasmic streaming compared to *A. cepa* (Figure 6B). After 16 h of dehydration, cell layers obtained from *A. fistulosum* had a 57.6% viability compared to 7.1% in *A. cepa* (Figure 6A). The trend of increased viability in *A. fistulosum* continued as the length of dehydration was extended. After 18 h, *A. fistulosum* epidermal cell layers had 39.2% viability while those obtained from *A. cepa* had a percent viability of 2.5% (Figure 6A).

In addition, there was a higher level of FDA-based “greenness” within calcium treated cell layers, indicating an increased level of viability (Figures S2 and S3). Generally, cell layers obtained from *A. fistulosum* also had a significantly (*p* < 0.05) increased level of greenness compared to those obtained from *A. cepa*, indicating greater viability following dehydration stress (Table S8). In addition, differences amongst other relationships were also found to be statistically significant (*p* < 0.05) (Table S8).

**Figure 7:**
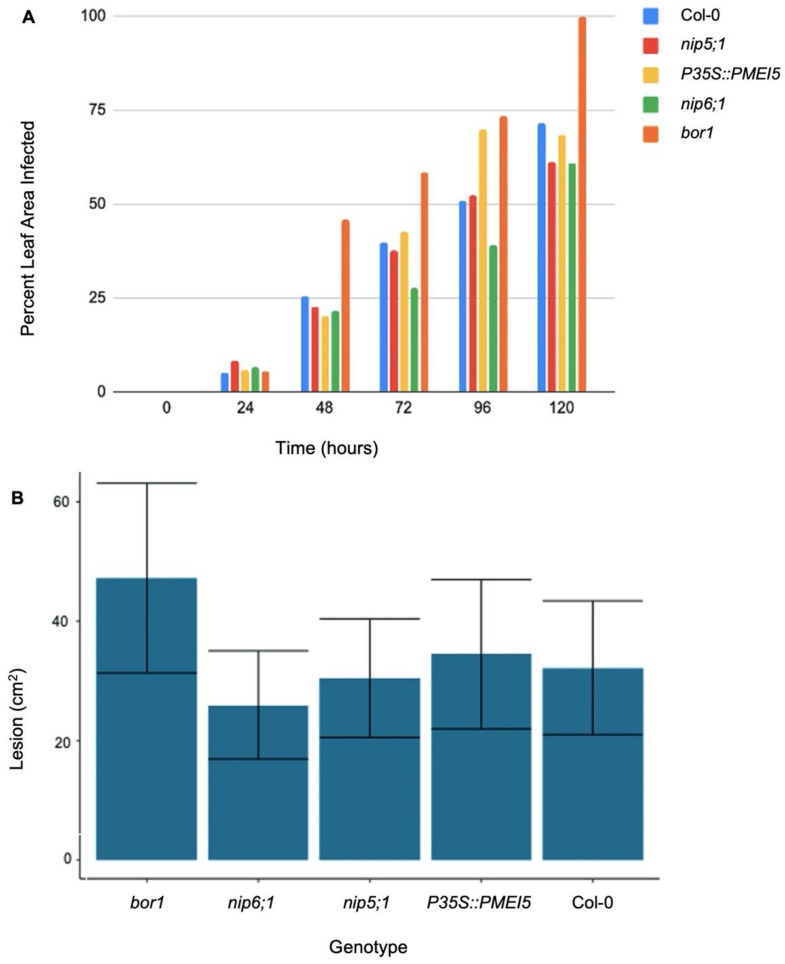
(**A**) Average lesion size on *Arabidopsis thaliana* leaves caused by *Botrytis cinerea*. Five genotypes were examined (*bor1* (boron transporter mutant), *nip5;1* (boron transporter mutant), *nip6;1* (boron transporter mutant), *p35S::PMEI5* (*PMEI* overexpression), and Col-0 (wild type)) over a 0–120 h period post-inoculation. (**B**) Boxplot showing average lesion size on *Arabidopsis thaliana* leaves caused by *Botrytis cinerea*. Five genotypes were examined (*bor1* (boron transporter mutant), *nip5;1* (boron transporter mutant), *bor1* (boron transporter mutant), *nip6;1* (boron transporter mutant), *p35S::PMEI5* (PMEI mutant), and Col-0 (wild type)) over a 0–120 h period post-inoculation. Error bars represent standard error. See Tables S9 and S10 for statistical analyses.

Boron also significantly (*p* < 0.05) reduced water loss in pure pectin solutions (Figure 4B). After 6 h, 8% GENU BETA pectin with boron (proxy for *Arabidopsis*) had a 59.7% water loss compared to 63.7% for the GENU BETA pectin control (Figure 4A). While similar dehydration stress tolerance trends were observed in *Arabidopsis* boron transporter mutants, the differences were non-significant (*p* > 0.05) (Figure 7B). Moreover, the over-expression of *PMEI5* did not significantly influence dehydration stress resistance (*p* > 0.05) (Figure 7B).

However, mutations in boron transporters and the over-expression of *PMEI5* were found to influence the rate of infection for both fungal pathogens (Figure 7). The analysis of the rate of *B. cinerea* infection amongst the genotypes of interest found *bor1* to be the most susceptible to the pathogen, consistently having the greatest percent area of infection from 1 dpi (days post-inoculation) (Figure 7). In general, the percentage of leaf area infected in the *bor1* leaves was significantly (*p* < 0.05) greater compared to all the other genotypes of interest (Figure 7B). At 5 dpi, 100% of the *bor1* leaf tissue was covered in a *B. cinerea* lesion (Figure 7A). By comparison, *nip6;1* had 60.7% of its leaf area infected, leaves obtained from *nip5;1* were 61.4% covered in a *B. cinerea* lesion, while *p35S::PMEI5* leaves were 68.4% infected (Figure 7A). Col-0 leaves were 71.4% covered by the lesion (Figure 7A).

**Figure 8:**
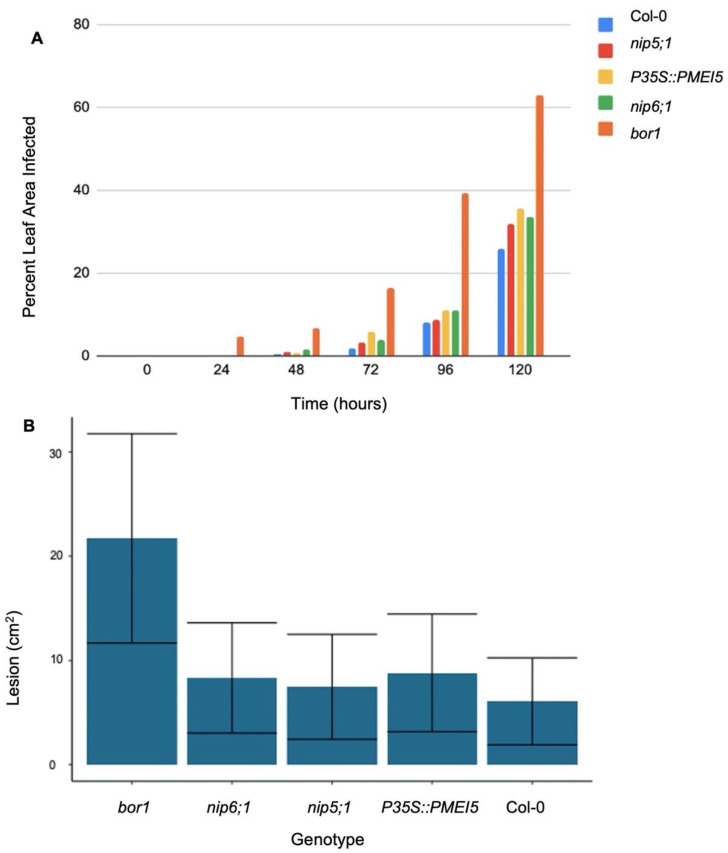
(**A**) Average lesion size on *Arabidopsis thaliana* leaves caused by *Colletotrichum higginsianum*. Five genotypes were examined (*bor1* (boron transporter mutant), *nip5;1* (boron transporter mutant), *bor1* (boron transporter mutant), *nip6;1* (boron transporter mutant), *p35S::PMEI5* (PMEI mutant), and Col-0 (wild type)) over a 0–120 h period post-inoculation. (**B**) Boxplot showing average lesion size on *Arabidopsis thaliana* leaves caused by *Botrytis cinerea*. Five genotypes were examined (*bor1* (boron transporter mutant), *nip5;1* (boron transporter mutant), *bor1* (boron transporter mutant), *nip6;1* (boron transporter mutant), *p35S::PMEI5* (PMEI mutant), and Col-0 (wild type)) over a 0–120 h period post-inoculation. Error bars represent standard error. See Tables S11 and S12 for statistical analyses.

In general, over the entire course of the infection, *bor1* was also significantly (*p* < 0.05) more susceptible to *C. higginsianum* compared to Col-0 (Figure 8B). At 5 dpi, over 60% of *bor1* leaf tissue was covered in a lesion caused by *C. higginsianum*, while all other genotypes of interest had less than 40% of their leaf tissue covered by a lesion (Figure 8A). At 1 dpi, the percentage leaf area covered by lesions from *C. higginsianum* was over 30% larger compared to the other genotypes analyzed (Figure 8A).

## 4. Discussion

### 4.1. Mechanical Changes and Calcium Localization

The structural role that pectin plays in the cell wall is critical to a plant’s ability to withstand stress [4]. Within the cell wall, pectin is far from the only critical component. However, what sets pectin apart from other components of the cell wall is the unique ability for HG and RG-II to form complex structures through cross-linkages to other elements [5,7]. Both the rheological results and those obtained from the analysis of shear force are suggestive that structural changes occurred because of calcium and boron application. Furthermore, the addition of these elements likely resulted in the formation of “egg-box” structures and RG-II dimers, as they are known to influence cell wall integrity, including rigidity and tensile strength [6,7,61].

The notion that calcium–HG cross-links form within *A. fistulosum* epidermal cell walls because of calcium application is further supported by images obtained from the Advanced Photon Source (Lemont, IL, USA) using the 20-ID beamline, the X-ray microprobe technique and X-ray fluorescence. These images clearly show an accumulation of calcium around the cell walls of epidermal cells obtained from calcium treated *A. fistulosum* and are a key piece of the puzzle in the narrative of the formation of “egg-box” structures. Within plant cells, pectin is only found within the cell wall and is most highly concentrated in the middle lamella and the primary cell wall [4]. Thus, the localization of calcium to the cell wall/middle lamella region and the increased shear force suggest that the application of calcium to *A. fistulosum* results in the formation of calcium–HG cross-links. Our next step was to analyze how the likely formation of these structures, in addition to boron and PMEIs, would influence dehydration stress resistance.

### 4.2. Dehydration Stress in Allium spp. and Pectin Proxy

In addition to causing structural changes within plant tissue, the formation of calcium–HG cross-linkages has also been tied to reduced cell wall permeability [12]. Thus, it was hypothesized the application of calcium would increase resistance to dehydration stress tolerance by reducing percentage water loss.

Even though calcium was found to significantly (*p* < 0.05) reduce percentage water loss in high methylated citrus pectin (proxy for *Allium*), the role calcium played on improving dehydration stress resistance in *A. fistulosum* and *A. cepa* was non-significant (*p* > 0.05) and is indicative of the complex nature of plants compared to pure systems. Nevertheless, previous studies have observed the efficacy of calcium application in enhancing drought stress tolerance/resistance in species such as *Beta vulgaris*, *Nicotiana tabacum* and wheat [62,63,64].

The discrepancy between these previous findings and those within this paper may be the result of a multitude of reasons, one of them being pore size. While the exact diameter of pores within the *A. fistulosum* epidermal cell layer is not known, previous research has found that, under cold acclimation, the diameter is reduced to less than 1.3 nm; however, the diameter of a water molecule at 25 °C is ~0.27 nm (2.7 Å) [65,66]. Therefore, even if permeability was reduced as a result of calcium application, the pore size was still larger than that of a water molecule. The large volume of water within the sheaths of *A. fistulosum* and *A. cepa* may also mask the effect of the calcium treatment.

Calcium is a complex element within plant systems, playing a role in a wide range of functions, including acting as a signaling molecule [67]. In its role as a signaling molecule, calcium has also been found to influence tolerance and resistance to drought stress in a variety of species [63,64,68]. For example, Knight et al. (1997) observed changes in the concentration of free calcium within cytosol during drought stress treatment in Arabidopsis [68]. More generally, the three main calcium sensors (1) calcineurin B-like protein; (2) calmodulin, and calmodulin-like proteins; and (3) calcium-dependent protein kinases) transduce Ca^2+^ signals, in turn causing the phosphorylation of a downstream target [69,70,71,72,73,74,75,76]. This ultimately results in a response to drought stress.

While calcium was unable (*p* > 0.05) to improve the overall resistance to dehydration in either *A. fistulosum* or *A. cepa*, *A. fistulosum* was significantly (*p* < 0.05) more tolerant to dehydration stress. *A. fistulosum* has also been found to be more tolerant to freezing-induced dehydration as compared with *A. cepa* [34,35]. *A. fistulosum* tolerates temperatures around −40 °C, while *A. cepa* lacks the ability to cold acclimate beyond −11 °C [34,35]. The connection between dehydration stress resistance and freezing stress tolerance is critical in the face of climate change. While initially drought stress and freezing stress may seem unrelated, both are tied to dehydration stress. During both drought stress and freezing stress, loss of turgor pressure can occur as the cell loses water [77,78,79]. As a result, cytorrhysis may occur beyond a tolerable limit. Oertli (1986) and Oertli et al. (1990) reported that the ability for the cell wall to resist cytorrhysis depends on the mechanical strength of the cell wall [80,81]. Therefore, the ability to resist dehydration stress and to tolerate freezing stress may be related to the mechanical strength of the wall within *A. fistulosum*. Pectin is known to play a large role in the strength and stability of the cell wall [4].

### 4.3. Dehydration Stress in Arabidopsis and Pectin Proxy

Similar to calcium and HG, boron and RG-II have the ability to form cross-linkages, which results in the formation of RG-II dimers [4,5,6]. Previous research by Panter et al. (2019) examining the *MUR1* gene in *Arabidopsis* found that the formation of these RG-II dimers improves tolerance to freezing stress [24]. Since dehydration stress is associated with freezing stress, it is plausible that RG-II dimers positively influence the water holding capacity of pectin. Thus, the addition of boron to GENU BETA likely resulted in the formation of RG-II dimers since the 8% GENU BETA pectin with boron lost significantly (*p* < 0.05) less water after 6 h compared to 8% GENU BETA pectin without boron.

Boron was also found to influence dehydration stress resistance in *Arabidopsis* in the various boron-transporter mutants. While none of the observed changes in dehydration stress resistance were found to be significant (*p* > 0.05) amongst the three boron-transporter mutants explored, *bor1* lost the most water. Decreased resistance to dehydration stress in *bor1* compared to *nip5;1* and *nip6;1* may be the result of differences in the roles of the boron transporters and further speaks to the importance of understanding the specific nature of the transporters.

Briefly, NIP5;1 and NIP6;1 are localized in the plasma membrane and are involved in borate transport [36,37]. NIP5;1 is also involved in arsenite transport [38]. In comparison, BOR1 is localized in the cytoplasm, endosome and vacuole in addition to the plasma membrane [38]. BOR1 is also involved in a wider range of functions, including detection of nutrients, ion homeostasis and transmembrane transport, as well as borate transport and response to boron-containing substances [38]. Therefore, a mutation in BOR1 may be more deleterious. However, there is a lack of additional research exploring NIP, BOR or other boron transporters in relation to dehydration stress resistance. Nonetheless, a range of species, including maize, wheat and tomatoes, had enhanced drought stress tolerance as a result of boron application [22,23,82,83]. This further implicates the importance of boron, and likely its structural role in the ability to retain water.

In addition to calcium and boron, PMEIs are another important aspect of pectin modifications and cell wall structure. PMEIs provide negative feedback on PMEs, in turn controlling the ability for “egg-box” structure formation in addition to altering the cell wall in a range of other structural ways [7]. The family of PMEIs within *Arabidopsis* is broad, and currently includes 71 putative genes [7,84,85]. Despite a lack of significance (*p* > 0.05), our results showed an over-expression of PMEI5 was beneficial in resistance to dehydration stress, by way of reducing percentage water loss and electrolyte leakage.

While seemingly counterintuitive, given that the over-expression of PMEI would reduce the formation of “egg-box” structures, research from An et al. (2008) supports our findings [86]. They found the over-expression of CaPMEI1, a PMEI from peppers, enhanced tolerance to drought stress [86]. This was attributable to an increased tolerance to oxidative stress, which was observed in the *CaPMEI1-OX Arabidopsis* line, which may in turn reduce the damage caused by stresses such as drought [86]. However, findings by An et al. (2008) and those observed in this study stand in direct contrast to findings by from Amsbury et al. (2016) and Yang et al. (2019), who found increased de-methylation of pectin enhanced drought stress tolerance [86,87,88]. This may be the result of differences amongst the various PMEIs.

### 4.4. Fungal Pathogens

Aside from abiotic stresses, biotic stresses also pose an immense threat to plants. Since both categories of stress may occur at the same time, a common mechanism of defense is critical to survival. The cell wall itself is the first line of defense and often serves as a signaling mechanism alerting plants to disease through pattern recognition receptors (PRRs) and damage-associated molecular patterns (DAMPs) [89]. Pectin methylesterase (PME) has long been associated with facilitated viral movement through the association between viral-encoded mobility proteins (MPs) and PME in which PME specifically recognizes binding domains of the viral MPs [90]. Lionetti et al. (2014) hypothesized once bound, the PME-MP increase the diameter of the plasmodesmata through the loosening of the callose and thereby facilitating greater viral movement [91]. They proposed that the inhibitor, PMEI, inhibits viral movement due to the reduction in PME-MP, thereby limiting the plasmodesmata dilation [91]. While the PMEI5 in our experiments did not show a significant reduction in fungal spread, it could be due to differences in homologous traits between the various PMEs and PMEIs. In Arabidopsis, there are 71 known PMEI genes and 66 known PME genes [7,85,92]. Given the large number of PMEs, it is highly plausible that only specific PMEs interact with MPs. The ability for the cell wall to remodel in response to viral pathogens is another critical component in defense, as it increases the strength of the cell wall [93]. This process has been found to be an important mechanism of defense against a variety of viral pathogens including halo blight disease in common bean, and *Potato virus* [93,94,95]. PMEI and PME have both been found to mediate cell wall remodeling [96,97]. While this set of experiments did not specifically focus on cell wall modeling in relation to viruses, the role of PMEI5 in connection with cell wall remodeling and defense against *B. cinerea* and *C. higginsianum* should be explored further. The analysis of lesion size over the course of 5 dpi for both *B. cinerea* and *C. higginsianum* demonstrated boron plays an integral role in resistance against both pathogens. *bor1* was significantly (*p* < 0.05) less resistant to *B. cinerea* compared to the other genotypes of interest (*nip5;1*, *nip6;1*, *p35S::PMEI5* and Col-0). This is likely the result of structural changes within pectin, and more specifically RG-II, given the findings of Petrasch et al. (2019) [28]. *bor1* was also significantly (*p* < 0.05) less resistant to *C. higginsianum* compared to Col-0. At 5 dpi, 100% of the total leaf area from *bor1* leaves was covered in a *B. cinerea* lesion. In comparison, leaves obtained from the other genotypes had less than 72% of their total leaf area covered lesions.

Since the BOR1 transporter is more widely expressed within the plant compared to NIP5;1 and NIP6;1, the *bor1* genotype likely has a lower concentration of boron [36,37,38]. Thus, the reduced resistance to *B. cinerea* in *bor1* compared to *nip5;1* and *nip6;1* may be the result of a lower concentration of boron, as boron has been previously found to mitigate *B. cinerea* infections [31].

Similarly, at 5 dpi following inoculation with *C. higginsianum*, 63.1% of the total *bor1* leaf area were covered in *C. higginsianum* lesion, compared to an average of only 31.7% for the other genotypes of interest. In addition to having twice the total leaf area covered in a lesion at 5 dpi, as early as 1 dpi, the *bor1* total leaf area covered in a *C. higginsianum* lesion was over 30 times greater compared to the other genotypes of interest, suggesting boron transported through the BOR1 channel is vital in the resistance to this disease. It is well understood that boron plays a critical role in the structure of the cell wall and a mutation in BOR1 would likely reduce the concentration of boron within the cell wall [98].

The reduced function of NIP5;1 and NIP6;1, in addition to the likelihood of redundancy within the NIP family, may be responsible for differences in leaf tissue infections compared to BOR1.

Furthermore, the over-expression of PMEI1, PMEI2, PMEI10, PMEI11 and PMEI12 has been previously found to help to maintain the integrity of the cell wall in plants infected with *B. cinerea* [26,27]. However, the over-expression of PMEI5 in the *p35S::PMEI5* did not significantly (*p* > 0.05) alter resistance to *B. cinerea*. Instead, leaves obtained from the *p35S::PMEI5* had the second greatest leaf area covered in *B. cinerea* lesions, after *bor1*. The contrasting nature of these findings compared to those obtained during our experiments may be the result of variation amongst PMEIs. PMEI5 belongs to Group 3 of PMEIs while previously explored PMEIs belong to other groups [26]. Future studies should focus on gaining a greater understanding into the differences between Group 1, 2 and 3 PMEIs.

Moreover, the more substantial lesion spread at 1 dpi on *bor1* leaves suggests weaker cell walls within the *bor1* genotype. *C. higginsianum* penetrates the cell wall of the host through physical pressure without the use of enzymes, unlike *B. cinerea* [99]. The rapid growth of lesions on *bor1* leaves is indicative that *C. higginsianum* may have penetrated the cell walls with less pressure, signaling potential weakness of the *bor1* cell walls, likely the result of lower concentrations of boron and in turn, a reduction in the number of RG-II dimers. Differences in function for the BOR1 transporter in comparison to NIP5;1 and NIP6;1 may reflect the variation observed in the percentage leaf area infected. More generally, while boron has been previously reported to act as an antifungal agent for a range of species infected with *Colletotrichum graminicola*, to our knowledge, there are no studies exploring NIP, BOR or any other B-transporters in relation to *C. higginsianum* [33,34].

## 5. Conclusions

While modifications in pectin do not allow the cell wall to form an impassable barrier, these elements remain critical in the role of the cell wall as a barrier to stress. While our findings on cell wall rheology, shear force, calcium localization and analysis of water loss in pure pectin standards are indicative that “egg-box” structures and RG-II dimers likely formed, this alone was not sufficient in significantly (*p* > 0.05) improving dehydration stress tolerance. Variation was also observed with respect to resistance to the fungal pathogens of interest, suggesting that while boron and PMEI5 likely play a role in resistance to dehydration stress and fungal pathogens, the true picture appears to be far more nuanced. Overall, our findings demonstrate the cell wall is not a silver bullet to dehydration stress or fungal pathogens. Even so, the results of this study help to further our understanding of the role of the cell wall as a physical barrier to both abiotic and biotic stresses.

## Data Availability

Data supporting the above findings are available within this paper and Supplementary Materials published online.

## Supplementary Materials

The following supporting information can be downloaded at: https://www.mdpi.com/article/10.3390/plants11030385/s1. Figure S1: Generalized additive models showing the relationship between temperature (°C) and pectin viscosity (Pa.S), Figure S2: Analysis of green pixels from images obtained from *Allium fistulosum* epidermal cell layers stained with fluorescein diacetate, Figure S3: Analysis of green pixels from images obtained from *Allium cepa* epidermal cell layers stained with fluorescein diacetate; Table S1: Primers used to genotype *nip5;1-1*, *nip6;1-2* and *bor1-3*, Table S2: ANOVA for generalized additive models examining the relationship between temperature (°C) and pectin viscosity (Pa.S), Table S3: ANOVA analyzing the force required to shear *Allium fistulosum* sheaths, Table S4: ANOVA analyzing percent water loss in *Allium fistulosum* and *Allium cepa* sheaths, Table S5: ANOVA analyzing percent water loss in pectin solutions, Table S6: Tukey’s test analyzing percent water loss in pectin solutions, Table S7: ANOVA examining percent viability based on electrolyte leakage following dehydration in *Allium fistulosum* and *Allium cepa*, Table S8: Two-sample *t*-tests analyzing the intensity of green pixels following staining with fluorescein diacetate in *Allium fistulosum* and *Allium cepa*, Table S9: ANOVA analyzing lesion size following *Botrytis cinerea* inoculation in *Arabidopsis*, Table S10: Tukey test analyzing lesion size following *Botrytis cinerea* inoculation in *Arabidopsis*, Table S11: ANOVA analyzing lesion size following *Colletotrichum higginsianum* inoculation in *Arabidopsis*, Table S12: Tukey test analyzing lesion size following *Colletotrichum higginsianum* inoculation in *Arabidopsis*.

## Abbreviations

AIC Score	Akaike Information Criterion (AIC). Used to select the optimal generalized additive model. A lower value indicates a more optimal model.
BOR1	Boric acid channel: part of the BOR family.
CA	Calcium Applied/ Calcium Treatment.
Cell Wall	Within the context of this paper, this term also incorporates the middle lamella.
EDF	Empirical Distribution Factor. Distribution of a function, where a value of one is indicative of a linear relationship, while a value above one is indicative of a non-linear relationship.
FDA	Fluorescein Diacetate.
GAM	Generalized Additive Model. A generalized linear model where the effect of predictive variables is captured using smoothing functions.
GB	GENU BETA Pectin (Sugar Beet Pectin).
HG.	Homogalacturonan.
HM	High Methylated Citrus Pectin.
LM	Low Methylated Citrus Pectin.
NCA	No Calcium Applied/ No Calcium Treatment.
NIP5:1	Boric acid channel, part of the NIP family.
NIP6:1	Boric acid channel, part of the NIP family.
PMEI5	Pectin methylesterase inhibitor five, part of the PMEI family.
RG-II	Rhamnogalacturonan II.
