# Supplementary material for: With a Little Help from My Cell Wall: Structural Modifications in Pectin May Play a Role to Overcome Both Dehydration Stress and Fungal Pathogens
Source: Plants (Basel). 2022 Jan 30;11(3):385. doi: 10.3390/plants11030385 (PMC8838300; doi:10.3390/plants11030385)
Supplement: Supplementary file 1 [file plants-11-00385-s001.zip › plants-1530735-supplementary.pdf]

Table S1 Primers used to genotype *nip5;1-1*, *nip6;1-2* and *bor1-3*

Line	SALK	Forward Primer	Reverse Primer
AT4G10380 (*nip5;1-1*)	SALK_122287C	TCCTAGCTCCATTTTCGTTTTC	CTCCAAGTGTGACGTAAACCC
AT1G80760 (*nip6;1-2*)	SALK_046323C	TGTTGGGACATTGATCCTGA	TCATCTTCTGAAGCTCCTC
AT2G47160 (*bor1-3*)	SALK_037312	ATGCTTGATGTTCCAATCGTC	ATCCATGTGAGACCAAAGCAG

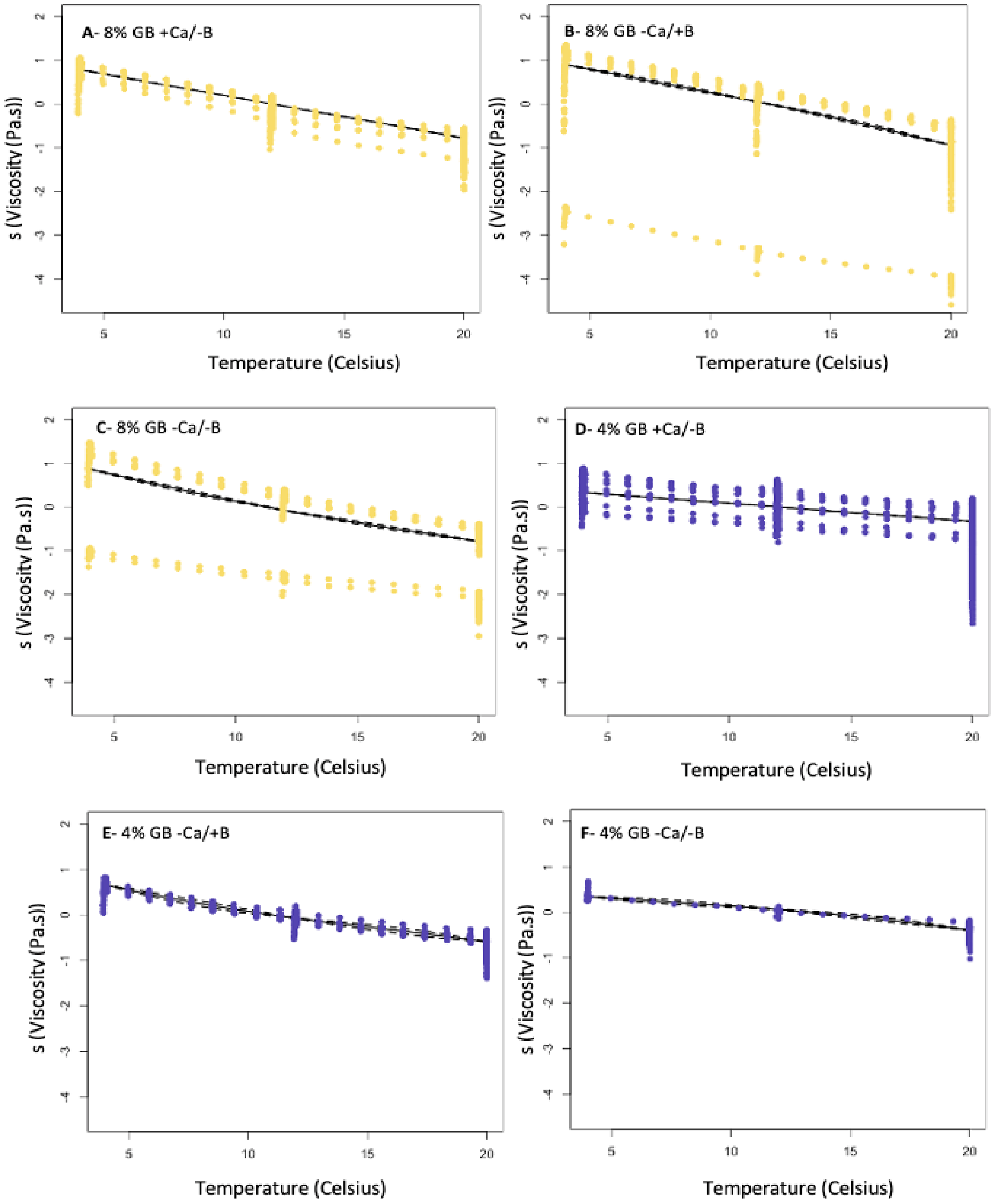

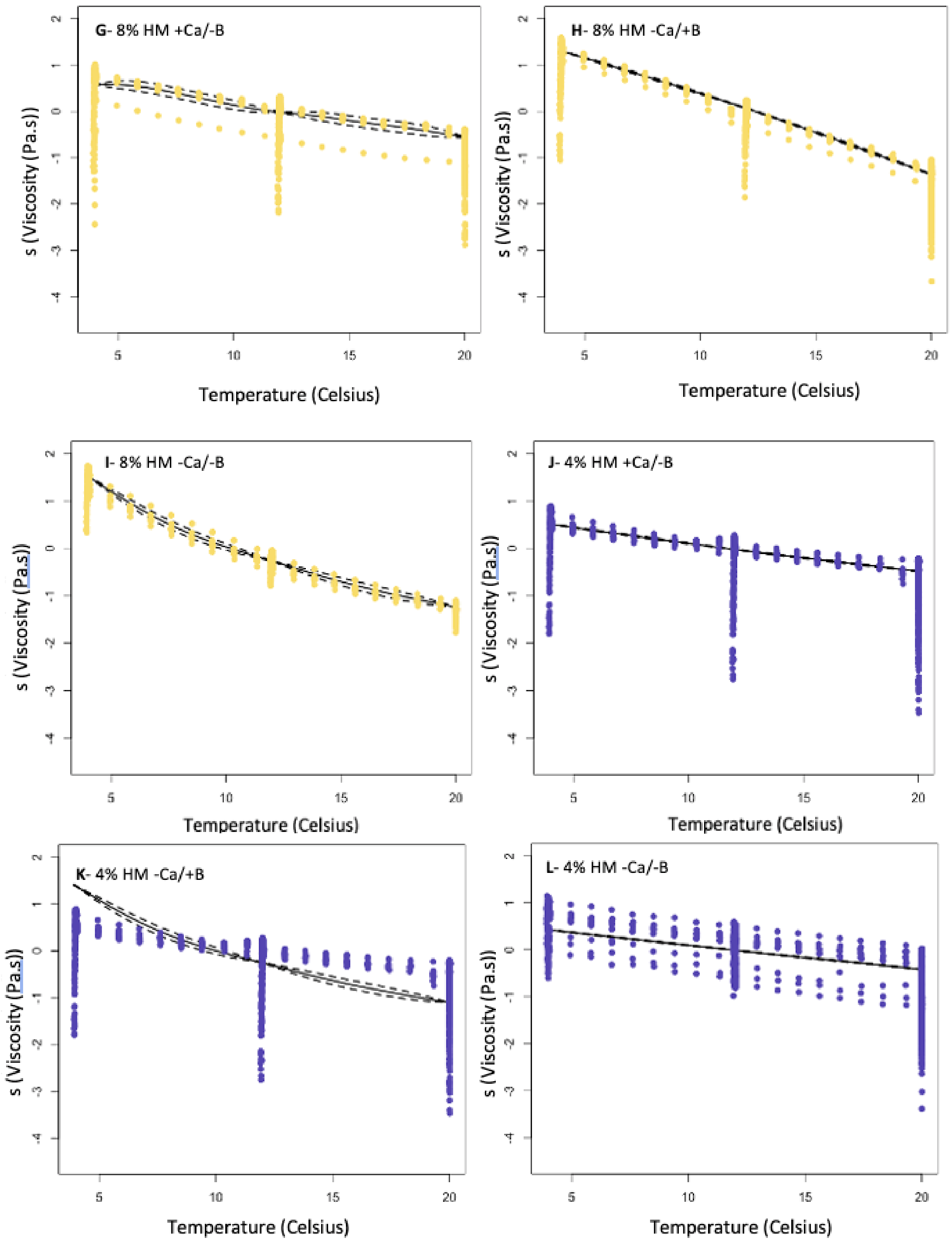

Figure S1. Generalized additive models which show the relationship between temperature (ºC) and viscosity (Pa.s) for each of the 12 pectin solutions (A-L) analyzed using a rheometer. Solid dark lines indicate the mean value for the response variable, while dotted lines show 95% confidence intervals around predicted values. Individual dots are represented single data points, which are an average across 12 replicates. Y-axis values were smoothed, as indicated by the "s".

Statistical analysis found significant differences between all pectin solutions and viscosity ($p<0.05$).

Table S2 ANOVA ran on a generalized additive model (GAM) examining pectin type, concentration, boron and calcium in combination with respect to relationship with temperature and viscosity

**Data Selection**	**Smoothing Effect**	**EDF**	**Ref.df**	**F-value**	**P-value**
Combined Treatment Groups	s(Temperature):8% GenuBeta -Ca / -B	1.996	2.004	143	<2e-16 ***
	s(Temperature):8% GenuBeta +Ca / -B	1.00	1.000	281	<2e-16 ***
	s(Temperature):8% GenuBeta -Ca / +B	2.722	2.923	136	<2e-16 ***
	s(Temperature):8% HighMethylated -Ca / -B	2.858	2.981	318	<2e-16 ***
	s(Temperature):8% HighMethylated +Ca / -B	8.925	8.998	145	<2e-16 ***
	s(Temperature):8% HighMethylated -Ca / +B	1.993	2.003	409	<2e-16 ***
	s(Temperature):4% GenuBeta -Ca / -B	2.825	2.970	223	<2e-16 ***
	s(Temperature):4% GenuBeta +Ca / -B	1.062	1.121	440	<2e-16 ***
	s(Temperature):4% GenuBeta -Ca / +B	2.645	2.874	559	<2e-16 ***
	s(Temperature):4% HighMethylated -Ca / -B	1.721	1.922	354	<2e-16 ***
	s(Temperature):4% HighMethylated +Ca / -B	1.959	1.999	608	<2e-16 ***
	s(Temperature):4% HighMethylated -Ca / +B	2.880	2.987	241	<2e-16 ***

Results from an ANOVA run on a GAM in which all 4 treatment options (pectin type, concentration, boron [B] and calcium [Ca]) were looked at in combination with respect to their relationship with temperature and effect on pectin viscosity. Two types of pectin were analyzed, Genu Beta and High Methylated. This GAM is representative of the lowest AIC value in the above table. * represents $p \leq 0.05$, ** represents $p \leq 0.01$, and *** represents $p \leq 0.001$. $p<0.05$ is significant.

Table S3 ANOVA analyzing the impact of calcium on the force required to shear *Allium fistulosum*

Data Selection	Df	Sum Sq.	Mean Sq.	F-value	P-value
Calcium	1	12019	12019	4.213	0.044*

Results from an ANOVA analyzing the force required to shear *Allium fistulosum* sheaths. * represents $p \leq 0.05$, ** represents $p \leq 0.01$, and *** represents $p \leq 0.001$. $p<0.05$ is significant.

Table S4 ANOVA examining overall percent water loss over 16-18hr in *Allium fistulosum* and *Allium cepa* (dehydration of sheath with attached cell layer)

Data Selection	Df	Sum Sq	Mean Sq	F-value	P-value
Species	1	41.8	41.8	8.635	0.043*
Calcium	1	1.1	1.1	0.236	0.653
Time	1	2452.4	2452.4	506.085	2.31e-05 ***
Species: Calcium	1	1.1	1.1	0.233	0.654
Species: Time	1	20.2	20.2	4.167	0.111
Calcium: Time	1	0.7	0.7	0.142	0.725
Species: Calcium: Time	1	0.6	0.6	0.124	0.742

Results from an ANOVA ran on data examining average percent water loss in 4cm sections of *Allium fistulosum* and *Allium cepa* sheaths with intact epidermal cell layers over 16-18hr. * represents $p \leq 0.05$, ** represents $p \leq 0.01$, and *** represents $p \leq 0.001$. $p<0.05$ is significant.

Table S5 ANOVA analyzing percent water loss in pectin solutions

Data Selection	Df	Sum Sq.	Mean Sq.	F-value	P-value
Pectin solutions	11	1199	109	49.53	< 2e-16 ***
Time	1	36953	36953	16787.40	< 2-16 ***
Pectin solutions: Time	11	302	27	12.46	8.4e-12 ***

Results from an ANOVA analyzing percent water loss over 6hr in pure pectin solutions. * represents $p \leq 0.05$, ** represents $p \leq 0.01$, and *** represents $p \leq 0.001$. $p<0.05$ is significant.

Table S6 Tukey test for ANOVA analyzing water loss in pectin solutions

**Treatment Combination**	**P value**
EightGB/NoCa/YesB-EightGB/NoCa/NoB	0.000 ***
EightGB/YesCa/NoB-EightGB/NoCa/NoB	0.041 *
EightHM/NoCa/NoB-EightGB/NoCa/NoB	0.999
EightHM/NoCa/YesB-EightGB/NoCa/NoB	0.319
EightHM/YesCa/NoB-EightGB/NoCa/NoB	0.000 ***
FouHM/YesCa/NoB-EightGB/NoCa/NoB	0.999
FourGB/NoCa/NoB-EightGB/NoCa/NoB	0.905
FourGB/NoCa/YesB-EightGB/NoCa/NoB	0.149
FourGB/YesCa/NoB-EightGB/NoCa/NoB	0.999
FourHM/NoCa/NoB-EightGB/NoCa/NoB	0.980
FourHM/NoCa/YesB-EightGB/NoCa/NoB	0.000 ***
EightGB/YesCa/NoB-EightGB/NoCa/YesB	0.000 ***
EightHM/NoCa/NoB-EightGB/NoCa/YesB	0.000 ***
EightHM/NoCa/YesB-EightGB/NoCa/YesB	0.000 ***
EightHM/YesCa/NoB-EightGB/NoCa/YesB	0.897
FouHM/YesCa/NoB-EightGB/NoCa/YesB	0.000 ***
FourGB/NoCa/NoB-EightGB/NoCa/YesB	0.000 ***
FourGB/NoCa/YesB-EightGB/NoCa/YesB	0.000 ***
FourGB/YesCa/NoB-EightGB/NoCa/YesB	0.000 ***
FourHM/NoCa/NoB-EightGB/NoCa/YesB	0.000 ***
FourHM/NoCa/YesB-EightGB/NoCa/YesB	0.000 ***
EightHM/NoCa/NoB-EightGB/YesCa/NoB	0.256
EightHM/NoCa/YesB-EightGB/YesCa/NoB	0.000 ***
EightHM/YesCa/NoB-EightGB/YesCa/NoB	0.000 ***
FouHM/YesCa/NoB-EightGB/YesCa/NoB	0.245
FourGB/NoCa/NoB-EightGB/YesCa/NoB	0.000 ***
FourGB/NoCa/YesB-EightGB/YesCa/NoB	0.000 ***
FourGB/YesCa/NoB-EightGB/YesCa/NoB	0.005 *
FourHM/NoCa/NoB-EightGB/YesCa/NoB	0.549
FourHM/NoCa/YesB-EightGB/YesCa/NoB	0.000 ***
EightHM/NoCa/YesB-EightHM/NoCa/NoB	0.056
EightHM/YesCa/NoB-EightHM/NoCa/NoB	0.000 ***
FouHM/YesCa/NoB-EightHM/NoCa/NoB	1.000
FourGB/NoCa/NoB-EightHM/NoCa/NoB	0.437
FourGB/NoCa/YesB-EightHM/NoCa/NoB	0.019 *
FourGB/YesCa/NoB-EightHM/NoCa/NoB	0.933
FourHM/NoCa/NoB-EightHM/NoCa/NoB	0.999
FourHM/NoCa/YesB-EightHM/NoCa/NoB	0.000 ***
EightHM/YesCa/NoB-EightHM/NoCa/YesB	0.000 ***
FouHM/YesCa/NoB-EightHM/NoCa/YesB	0.059
FourGB/NoCa/NoB-EightHM/NoCa/YesB	0.997

FourGB/NoCa/YesB-EightHM/NoCa/YesB	0.999
FourGB/YesCa/NoB-EightHM/NoCa/YesB	0.783
FourHM/NoCa/NoB-EightHM/NoCa/YesB	0.014 *
FourHM/NoCa/YesB-EightHM/NoCa/YesB	0.238
FouHM/YesCa/NoB-EightHM/YesCa/NoB	0.000 ***
FourGB/NoCa/NoB-EightHM/YesCa/NoB	0.000 ***
FourGB/NoCa/YesB-EightHM/YesCa/NoB	0.000 ***
FourGB/YesCa/NoB-EightHM/YesCa/NoB	0.000 ***
FourHM/NoCa/NoB-EightHM/YesCa/NoB	0.000 ***
FourHM/NoCa/YesB-EightHM/YesCa/NoB	0.000 ***
FourGB/NoCa/NoB-FouHM/YesCa/NoB	0.452
FourGB/NoCa/YesB-FouHM/YesCa/NoB	0.020 *
FourGB/YesCa/NoB-FouHM/YesCa/NoB	0.939
FourHM/NoCa/NoB-FouHM/YesCa/NoB	0.999
FourHM/NoCa/YesB-FouHM/YesCa/NoB	0.000 ***
FourGB/NoCa/YesB-FourGB/NoCa/NoB	0.966
FourGB/YesCa/NoB-FourGB/NoCa/NoB	0.999
FourHM/NoCa/NoB-FourGB/NoCa/NoB	0.183
FourHM/NoCa/YesB-FourGB/NoCa/NoB	0.021 *
FourGB/YesCa/NoB-FourGB/NoCa/YesB	0.534
FourHM/NoCa/NoB-FourGB/NoCa/YesB	0.004 *
FourHM/NoCa/YesB-FourGB/NoCa/YesB	0.457
FourHM/NoCa/NoB-FourGB/YesCa/NoB	0.700
FourHM/NoCa/YesB-FourGB/YesCa/NoB	0.001 **
FourHM/NoCa/YesB-FourHM/NoCa/NoB	0.000 ***

Results from a Tukey test conducted to analyze the relationship between pectin solutions of interest and percent water loss over 6hr. * represents $p \leq 0.05$, ** represents $p \leq 0.01$, and *** represents $p \leq 0.001$. $p<0.05$ is significant.

Table S7 ANOVA examining percent viability based on electrolyte leakage in *Allium fistulosum* and *Allium cepa* following 16-18hr dehydration of sheath

**Data Selection**	**Df**	**Sum Sq**	**Mean Sq**	**F-value**	**P-value**
Calcium	1	53	53	0.987	0.377
Time	1	1503414617	14617	272.916	7.86e-05 ***
Species	1	2530	2530	47.235	0.002 **
Calcium: Time	1	32	32	0.594	0.484
Calcium: Species	1	69	69	1.282	0.321
Time: Species	1	1183	1183	22.094	0.009 **
Species: Calcium: Time	1	36	36	0.670	0.459

Results from an ANOVA ran on data examining average percent viability in *Allium fistulosum* and *Allium cepa* epidermal cell layers following long term dehydration (16-

18hr) of the sheath with the epidermal cell layer intact. * represents $p \leq 0.05$, ** represents $p \leq 0.01$, and *** represents $p \leq 0.001$. $p<0.05$ is significant.

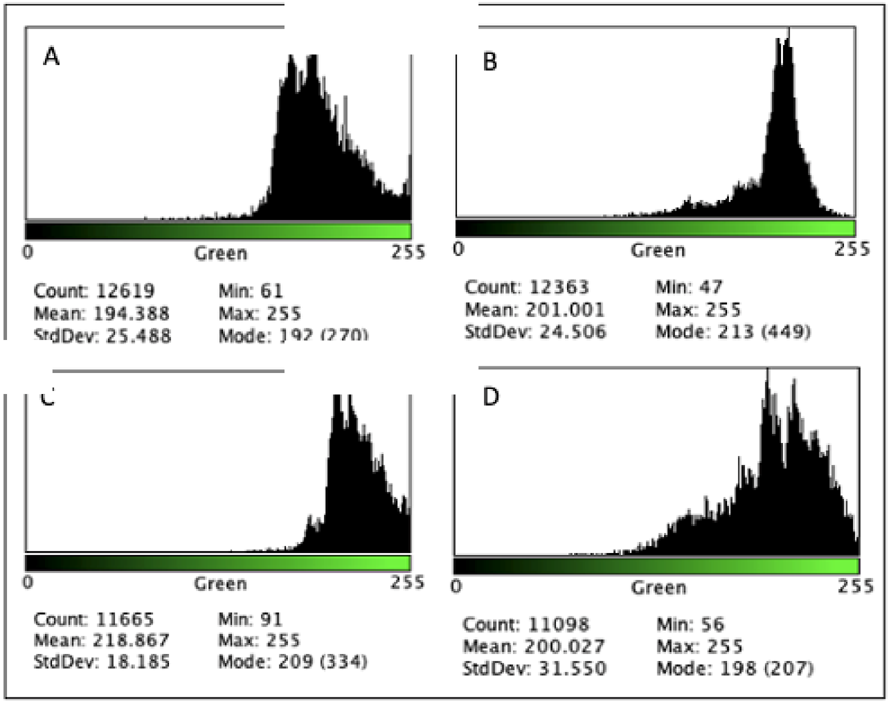

Figure S2 Analysis of green pixels from images of *Allium fistulosum* epidermal cell layers stained with fluorescein diacetate and imaged under fluorescence microscopy

Analysis of green pixels from images of *Allium fistulosum* epidermal cell layers stained with fluorescein diacetate following dehydration (16hr and 18hr) and subsequent rehydration (24hr). Images were taken using fluorescence microscopy within a LEICA DM4 B microscope. Image analysis was performed using ImageJ (Version 1.53a). Graphs from left to right: A). Post-16hr dehydration, NCA (-calcium), B). Post 16-hr dehydration, CA (+calcium), C). Post-18hr dehydration, NCA (- calcium), and D). Post 18-hr dehydration, CA (+calcium). Graphs were generated using ImageJ (Version 1.53a). See Table S7 for statistical analysis.

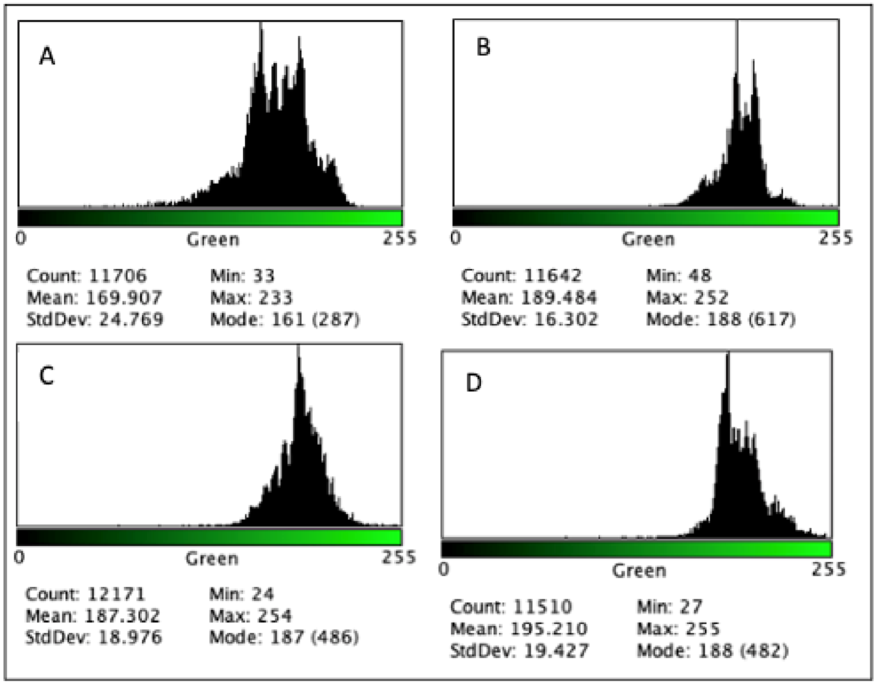

Figure S3 Analysis of green pixels from images of *Allium cepa* epidermal cell layers stained with fluorescein diacetate and imaged under fluorescence microscopy

Analysis of green pixels from images of *Allium cepa* epidermal cell layers stained with fluorescein diacetate following dehydration (16hr and 18hr) and subsequent rehydration (24hr). Images were taken using fluorescence microscopy within a LEICA DM4 B microscope. Image analysis was performed using ImageJ (Version 1.53a). Graphs from left to right: A). Post-16hr dehydration, NCA (-calcium), B). Post 16-hr dehydration, CA (+calcium), C). Post-18hr dehydration, NCA (- calcium), and D). Post 18-hr dehydration, CA (+calcium). Graphs were generated using ImageJ (Version 1.53a). See Table S7 for statistical analysis.

Table S8 Two-sample *T*-tests analyzing differences in green pixel intensity following fluorescein diacetate staining

**Groups Compared**	**T value**	**Df**	**P-value**
A. fistulosum vs. *A. cepa*	21.506	1	0.029*
NCa vs. Ca *A. fistulosum* (16hr)	59.817	1	0.011*
NCa vs. Ca *A. fistulosum* (18hr)	22.198	1	0.029*
NCa vs. Ca *A. cepa* (16hr)	18.364	1	0.035*
NCa vs. Ca *A. cepa* (18hr)	48.358	1	0.013*

Ca *A. fistulosum* vs. Ca *A. cepa* (16hr)	33.896	1	0.019*
NCa *A. fistulosum* vs. NCa *A. cepa* (16hr)	14.882	1	0.043*
Ca *A. fistulosum* vs. Ca *A. cepa* (18hr)	48.358	1	0.013*
NCa *A. fistulosum* vs. NCa *A. cepa* (18hr)	12.866	1	0.049*

Results from *t*-tests ran comparing epidermal cell layers obtained from *Allium fistulosum* and *Allium cepa* epidermal sheaths following dehydration over 16-18hr and subsequent rehydration. CA represents calcium treated plants, while NCA corresponds to plants that did not receive calcium.

Table S9 ANOVA analyzing average lesion size over 5 days following *Botrytis cinerea* inoculation in *Arabidopsis thaliana*

	**Df**	**Sum**	**Mean sq**	**F value**	**P value**
Genotype	4	1533	383	11.636	4.81e-05 ***
Time	1	19926	19926	604.866	<2e-16 ***
Genotype: time	4	808	202	6.131	0.002 **

Results from an ANOVA examining average lesion size over 5 days following inoculation with B. cinerea into various *Arabidopsis thaliana* genotypes. Five genotypes were observed: Col-0, *nip5;1*, *nip6;1*, *p35S::PMEI5* and *bor1*. * represents $p \leq 0.05$, ** represents $p \leq 0.01$, and *** represents $p \leq 0.001$. $p<0.05$ is significant.

Table S10 Tukey test for results obtained from an ANOVA analyzing average lesion size over 5 days following *Botrytis cinerea* inoculation in *Arabidopsis thaliana*

**Data Selection**	**diff**	**lwr**	**upr**	**p adj**
nip6;1 & bor1	-21.257	-31.173	-11.341	0.000***
nip5;1 & bor1	-16.787	-26.703	-6.871	0.001***
p35S::PMEI5 & bor1	-12.763	-22.679	-2.847	0.008**
Col-0 *& bor1*	-15.028	-24.943	-5.112	0.002**
nip5;1 & nip6;1	4.469	-5.446	14.386	0.665
p35S::PMEI5 & nip6;1	8.494	-1.422	18.410	0.116
Col-0 *& nip6;1*	6.229	-3.686	16.146	0.359
p35S::PMEI5 & nip5;1	4.024	-5.892	13.940	0.743
Col-0 *& nip5;1*	1.759	-8.156	11.676	0.983
Col-0 *& p35S::PMEI5*	-2.264	-12.180	7.652	0.958

Results from a Tukey test examining average lesion size over 5 days following inoculation with B. cinerea into various *Arabidopsis thaliana* genotypes. Five genotypes were observed: Col-0, *nip5;1*, *nip6;1*, *p35S::PMEI5* and *bor1*. * represents $p \leq 0.05$, ** represents $p \leq 0.01$, and *** represents $p \leq 0.001$. $p<0.05$ is significant.

Table S11 ANOVA analyzing average lesion size over 5 days following *Colletotrichum higginsianum* inoculation in *Arabidopsis thaliana*

**Data Selection**	**Df**	**Sum**	**Mean sq**	**F value**	**P value**
Genotype	4	973	243	3.506	0.0252 *
Time	1	4043	4043	58.257	2.39e-07 ***
Genotype: time	4	679	170	2.445	0.080

Results from an ANOVA examining average lesion size over 5 days following inoculation with *Colletotrichum higginsianum* into various *Arabidopsis thaliana* genotypes. Five genotypes were observed: Col-0, *nip5;1*, *nip6;1*, *p35S::PMEI5* and *bor1*. * represents $p \leq 0.05$, ** represents $p \leq 0.01$, and *** represents $p \leq 0.001$. $p<0.05$ is significant.

Table S12 Tukey test for results obtained from an ANOVA analyzing average lesion size over 5 days following *Colletotrichum higginsianum* inoculation in *Arabidopsis thaliana*

**Data Selection**	**diff**	**lwr**	**upr**	**p adj**
nip6;1 & bor1	-13.399	-27.790	0.993	0.076
nip5;1 & bor1	-14.249	-28.641	0.143	0.053
p35S::PMEI5 & bor1	-12.906	-27.298	1.486	0.092
Col-0 *& bor1*	-15.643	-30.035	1.251	0.029 *
nip5;1 & nip6;1	-0.850	-15.241	13.542	0.999
p35S::PMEI5 & nip6;1	0.493	-13.899	14.885	0.999
Col-0 *& nip6;1*	-2.245	-16.636	12.147	0.989
p35S::PMEI5 & nip5;1	1.343	-13.049	15.735	0.998
Col-0 *& nip5;1*	-1.394	-15.786	12.997	0.998
Col-0 *& p35S::PMEI5*	-2.737	-17.129	11.654	0.978

Results from a Tukey test examining average lesion size over 5 days following inoculation with *Colletotrichum higginsianum* into various *Arabidopsis thaliana* genotypes. Five genotypes were observed: Col-0, *nip5;1*, *nip6;1*, *p35S::PMEI5* and *bor1*. * represents $p \leq 0.05$, ** represents $p \leq 0.01$, and *** represents $p \leq 0.001$. $p<0.05$ is significant.
